# INFLUENCE OF POSTTRAUMATIC STRESS AND ABNORMAL SPIROMETRY ON COGNITIVE PERFORMANCE IN 9/11 WTC RESPONDERS

**DOI:** 10.1093/geroni/igac059.1509

**Published:** 2022-12-20

**Authors:** Andrea Zammit, Charles Hall, Sean Clousten, Benjamin Luft

**Affiliations:** Rush Alzheimer's Disease Center, Chicago, Illinois, United States; Albert Einstein College of Medicine, Bronx, New York, United States; Stony Brook University, Stony Brook, New York, United States; Stony Brook University, Stony Brook, New York, United States

## Abstract

Post-traumatic stress disorder (PTSD) and abnormal spirometry are highly prevalent mental and health conditions in World Trade Center (WTC) responders. We tested the hypothesis that PTSD symptomatology and abnormal spirometry are conjointly and synergistically associated with poorer cognitive performance. A total of 1,326 responders (mean age = 53.1, SD = 8.1, 92% males) from the WTC Health Program took part in the study. PTSD symptomatology was assessed using the PCL-IV, and we calculated the FEV1/FVC ratio to measure pulmonary function. Cogstate assessments measured cognitive performance. Linear regressions were employed to evaluate PTSD and pulmonary function on cognitive performance while adjusting for age, sex, education, smoking status, and comorbidity. Higher PTSD symptomatology and lower pulmonary function were independently and conjointly negatively associated with cognitive performance. Further, a significant synergistic effect was present in that higher severity of PTSD symptomatology in the presence of lower pulmonary function was associated with poorer cognitive performance (estimate = -0.096, SE = 0.03, p <0.001). Results suggested that chronic stress and lung damage might share underlying biological mechanisms, including inflammatory and oxidative stress pathways, which may also be affecting the brain. Early intervention efforts to mitigate preventable cognitive decline in high-risk populations should be studied.

